# ZnS-Based Electrode Materials for Electrochemical Sensing (Environmental Monitoring and Food Samples) and Energy Storage Applications

**DOI:** 10.3390/bios15110730

**Published:** 2025-11-02

**Authors:** Chellakannu Rajkumar, Shanmugam Vignesh, Khursheed Ahmad, Tae Hwan Oh

**Affiliations:** School of Chemical Engineering, Yeungnam University, Gyeongsan 38541, Republic of Korea

**Keywords:** ZnS, composites, biosensors, energy storage, supercapacitors

## Abstract

In the present scenario, it is believed that the fabrication of cost-effective and environmentally friendly nanomaterials is of great significance for various optoelectronic and electrochemical applications. In the past few years, zinc sulfide and its composites with carbon-based materials, metal oxides, MXenes, metal–organic frameworks (MOFs) and other materials have been prepared for electrochemical applications. The ZnS-based materials exhibit good specific surface area, catalytic activity, and decent conductivity, which makes them promising materials for sensors and supercapacitors (SCs). In this review article, we briefly discuss the synthesis of ZnS using various methods, such as hydrothermal, microwave, sol–gel, electrochemical, and ultrasonication methods. Furthermore, ZnS and its composites for electrochemical sensors are reviewed. The limits of detection, sensitivity, stability, and selectivity of the reported sensors are discussed. Furthermore, studies based on ZnS and its composites for SC applications are reviewed. It was found that ZnS-based composites exhibit good electrochemical performance for SCs. The limitations and prospects of ZnS-based materials are also discussed. We believe that the present review article may be useful for researchers who are involved in the fabrication of ZnS-based materials for SCs and electrochemical sensing applications.

## 1. Introduction

In the current scenario, environmental pollution and the energy crisis are the two major concerns for the next-generation world [1,2]. Globalization and industrialization may increase environmental pollution, and energy demand is also continuously increasing with the increasing global population [3,4]. Therefore, it should be kept in mind that the future world may need to contend with these two major challenges. There are numerous environmental pollutants, which may come out of industries and pharmaceutical companies as effluents and pollute the groundwater and environment [5,6,7]. These pollutants may also affect human health and aquatic life [8,9]. Thus, monitoring environmental pollutants is of great significance. In this regard, conventional methods, such as photoluminescence [10], high-performance liquid chromatography (HPLC) [11], mass spectrometry [12], fluorescence [13], flow injection [14], and capillary electrophoresis [15], etc., were explored for the monitoring of various analytes. Unfortunately, these conventional approaches suffer from lack of portability, high operational time, requirement for highly qualified handlers, and high cost [16,17]. Therefore, some other, non-traditional methods should be explored for the determination of environmental pollutants.

In recent years, electrochemical methods have received enormous interest from the scientific community due to their simple operational principles, high sensitivity, decent selectivity, cost-effectiveness, and simple fabrication processes [18]. Electrochemical methods can be used for the determination of environmental pollutants and other biomolecules using a computer-controlled potentiostat system. Differential pulse voltammetry (DPV) [19], linear sweep voltammetry (LSV) [20], cyclic voltammetry (CV) [21], square wave voltammetry (SWV) [22], square wave anodic stripping voltammetry (SWASV) [23], and amperometry/chroamperometry (Amp/CA) [24,25] are widely used for the determination of targeted analytes through electrochemical methods. The sensitivity and selectivity of the electrochemical sensors are greatly influenced by the physicochemical characteristics of the electrocatalyst present on the working electrode surface. Therefore, it is believed that electrocatalysts with larger surface areas and high conductivities may exhibit high sensitivity for targeting analytes.

The energy crisis has led researchers to develop energy storage devices such as batteries and supercapacitors (SCs) [26,27]. SCs have emerged as one of the most promising energy storage systems and have the potential to bridge the gap between batteries and conventional capacitors [28]. SCs also offer high power density, decent specific capacitance, and longer cycle life [29]. The design and fabrication of novel electrode materials for SC applications are of great significance. Recent years have witnessed growth in the fabrication of numerous materials, such as metal oxides [30], polymers [31], metal sulfides [32], carbon-based materials [33], MXenes [34], and metal–organic frameworks (MOFs) [35], for SC applications. In particular, zinc sulfide (ZnS) is one of the most promising metal sulfides and has excellent electrochemical properties, with sufficient conductivity and surface properties for various applications [36,37,38]. Although other metal sulfides such as nickel sulfide (NiS), cobalt sulfide (CoS), and molybdenum disulfide (MoS_2_) are also promising materials for electrochemical applications [32], ZnS has several advantages, which make it a promising candidate for electrochemical applications. ZnS is an environmentally friendly metal sulfide, which has decent stability in alkaline media for electrochemical applications [27]. ZnS also shows corrosion resistance and long-term cyclic stability for SCs. Therefore, it is suggested that ZnS is a more efficient electrode material compared to other metal sulfides.

**Scheme 1:**
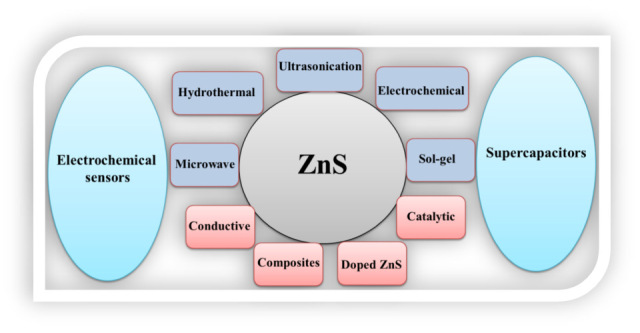
Schematic picture shows the properties and application of ZnS materials for electrochemical sensors and SCs.

In recent years, ZnS has been extensively used, whether in pristine form or in a hybrid composite form, as an electrode material for SC applications [39,40]. Herein, we report a review article on ZnS-based materials for electrochemical sensors and SC applications. We believe that the present review article may be useful for audiences working on electrochemical sensors and SC applications, as shown in Scheme 1.

## 2. Synthetic Methods

In previous years, various methods were extensively employed for the preparation of ZnS and its composites. These methods include the hydrothermal/solvothermal method, sol–gel method, electrochemical method, ultrasonication method, and microwave method. In this section, we briefly describe some of the reported synthesis methods for the preparation of ZnS and its composites.

### 2.1. Hydrothermal Method

The hydrothermal method is a widely used approach for the preparation of nanostructured materials. The hydrothermal method has several advantages such as pH, temperature, and time control. In this connection, Ibupoto et al. [41] also adopted the hydrothermal method for the preparation of ZnS nanostructures. Fluorine-doped tin oxide (FTO) was used as a glass substrate and washed with isopropanol (IPA) and dried. The zinc acetate dehydrate and thiourea solutions were prepared, and CTAB was also added to the reaction mixture. The FTO-based substrate was affixed in the Teflon-lined autoclave reactor containing the reaction mixture and sealed. The reaction was carried out at 200 °C for 12 h in an electric oven, which yielded a ZnS nanostructured material. Gao et al. [42] also adopted the hydrothermal method for the preparation of ZnS. Pink ZnS was obtained using zinc nitrate hexahydrate and thiourea as precursors. The reaction temperature was optimized, and the authors obtained a pink ZnS sample under optimized conditions. The authors also observed that the prepared ZnS had Zn vacancies. The above reports suggest that the hydrothermal method is promising to tune the surface morphological properties of the nanomaterials.

### 2.2. Microwave Method

It is well known that the microwave approach is an efficient method for the synthesis of nanomaterials. This method offer several advantages, including minimal solvent requirements, higher yield, enhanced product quality, and reduced reaction time. Ruiz et al. [43] adopted the microwave method for the formation of pristine ZnS and iron-doped ZnS materials. Typically, the authors used zinc sulfate heptahydrate solution and sodium sulfide as precursors. The Teflon vessel containing the reaction mixture was heated at 140 °C for 15 min under microwave irradiation. Wang et al. [44] also explored the microwave method for the synthesis of metal-doped ZnS for photocatalytic applications. The authors used a temperature of 120 °C for 10 min for the fabrication of ZnS, indicating that the microwave method can be used for the preparation of ZnS with a short reaction time.

### 2.3. Sol–Gel Method

The sol–gel method is a simple and energy-efficient approach, which has several other advantages, such as control of the pH and temperature. This method is also used to obtain films and powder, etc. In 1997, Stanić et al. [45] explored the sol–gel approach for the formation of ZnS. The authors used zinc tert-butoxide and hydrogen sulfide as precursors. Yang et al. [46] also used the sol–gel method for the formation of ZnS-based material. The authors prepared ZnS@N, an S-doped carbon nanosheet (ZnS@NSC) composite. The thiourea, glucose and zinc nitrate were used as precursors, and a high temperature of 600 to 800 °C was used for the annealing process. The major concern with the sol–gel method is the use of high temperature during the annealing process.

### 2.4. Electrochemical Synthesis Method

Hamla et al. [47] used the electrochemical method for the preparation of ZnS. The authors used zinc chloride (ZnCl_2_), sodium thiosulphate (Na_2_S_2_O_3_), and trisodium citrate (Na_3_C_6_H5O_7_) as precursors for the preparation of ZnS. Samskruthi et al. [48] also reported the fabrication of a ZnS/In_2_S_3_ composite using the electrochemical method. It is worth stating that the electrochemical method has several advantages, such as cost-effectiveness, simplicity and low-temperature processing. The electrochemical method is environmentally friendly and can be explored for the preparation of thin films for various electrochemical applications.

### 2.5. Ultrasonication Method

Previously, Xu et al. [49] adopted the sonication method for the preparation of ZnS nanoparticles (NPs). In another report, Annalakshmi et al. [50] also used the sonication method for the preparation of ZnS for the sensitive detection of carcinogenic nitrite ions. It can be stated that the ultrasonication method is also a cost-effective and facile approach for the preparation of nanomaterials. The sonication method is also considered a green synthesis method and can be used for the preparation of nanomaterials in bulk. The advantages and disadvantages of the different synthesis methods are summarized in Table 1.

**Scheme 2:**
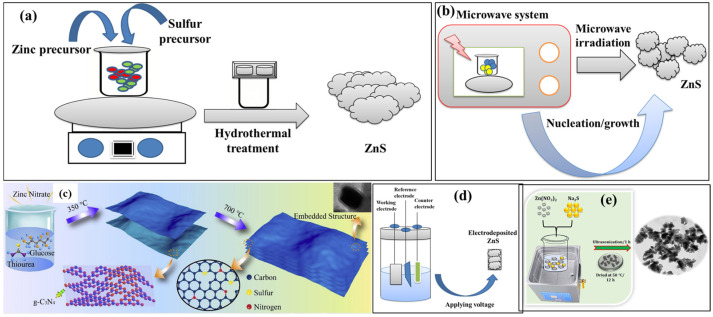
Schematic representation of the preparation of ZnS using (**a**) hydrothermal, (**b**) microwave, (**c**) sol–gel, (**d**) electrochemical and (**e**) ultrasonication methods. Reproduced with permissions [50].

General schematic pictures for the preparation of ZnS using different methods are included in Scheme 2. Scheme 2a shows the synthesis process for ZnS formation. Scheme 2b,c represent the preparation of the ZnS and ZnS composites using the microwave and sol–gel methods, respectively. Scheme 2d represents the preparation of ZnS using the electrochemical method, whereas Scheme 2e shows the formation of ZnS through the ultrasonication method.

## 3. ZnS in Electrochemical Sensors

**Figure 1:**
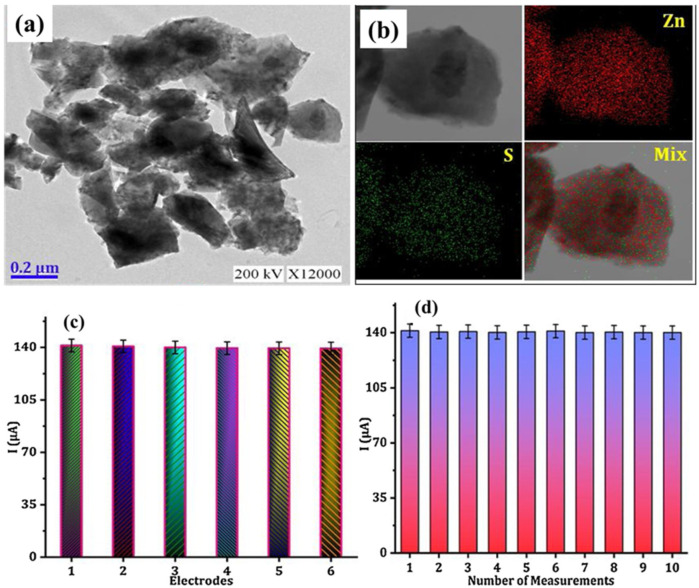
(**a**) TEM and (**b**) EDX mapping image of the prepared ZnS. (**c**) Reproducibility and (**d**) repeatability for nitrite detection. Reproduced with permission [50].

In previous years, numerous reports explored the use of ZnS nanoparticles (NPs) and composites for the development of electrochemical sensors. In this connection, Annalakshmi et al. [50] reported the synthesis of ZnS using the ultrasonication method. Figure 1a shows a transmission electron microscopic image of the prepared ZnS. The TEM image reveals that the obtained ZnS consists of a flake-like structure. The energy-dispersive X-ray spectroscopic (EDX) image revealed the uniform distribution of the Zn and S elements (Figure 1b). The ZnS-based electrochemical sensor was fabricated, and electrochemical studies exhibited a limit of detection (LOD) of 8.5 nM for nitrite detection using the amperometric technique. This sensor also indicated decent reproducibility and repeatability, as shown in Figure 1c,d, respectively.

**Figure 2:**
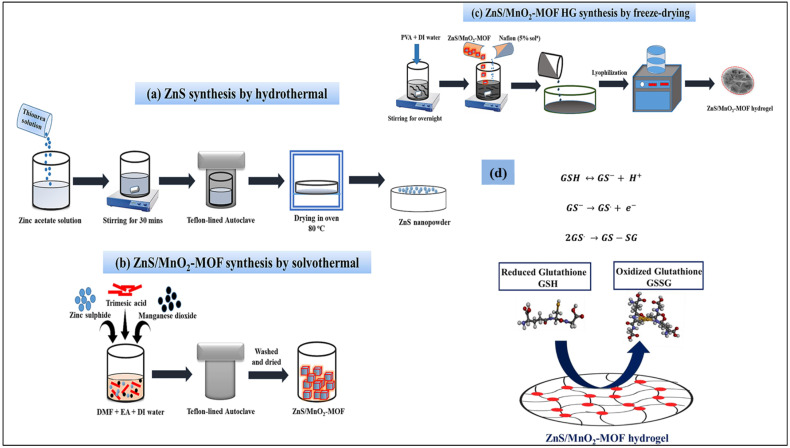
Schematic picture illustrates the formation of (**a**) ZnS, (**b**) ZnS/MnO_2_ and (**c**) ZnS/MnO_2_-MOF hydrogel. (**d**) Reaction process for GSH detection. Reproduced with permission [53].

Elnouby et al. [51] reported the preparation of ZnS using the ball mill approach. The X-ray diffraction (XRD) studies revealed that the prepared ZnS has a rhombohedral system with a polycrystalline nature. The mean crystallite size of the prepared ZnS was found to be 1.56 nm. The prepared ZnS NPs were explored for electrochemical sensing applications. The ZnS nanostructure-based electrochemical sensor was also developed for the detection of uric acid (UA) [52]. The ZnS urchin-like nanostructure-modified indium tin oxide (ITO) demonstrated improved sensitivity compared to the ZnS NPs or ZnS nanoflake-based electrodes. This may be attributed to the high surface area of the ZnS urchin-like nanostructures. This study proposed that the morphological features of the electrode materials may significantly influence the electrochemical performance of the sensors. In another previous work [53], a glutathione (GSH) electrochemical sensor was developed by employing a benign approach. In this connection, ZnS was combined with a manganese dioxide (MnO_2_)-metal–organic framework hydrogel (MOF-HG) using the lyophilization approach. The preparation of the electrode materials is described in Figure 2a–c.

**Figure 3:**
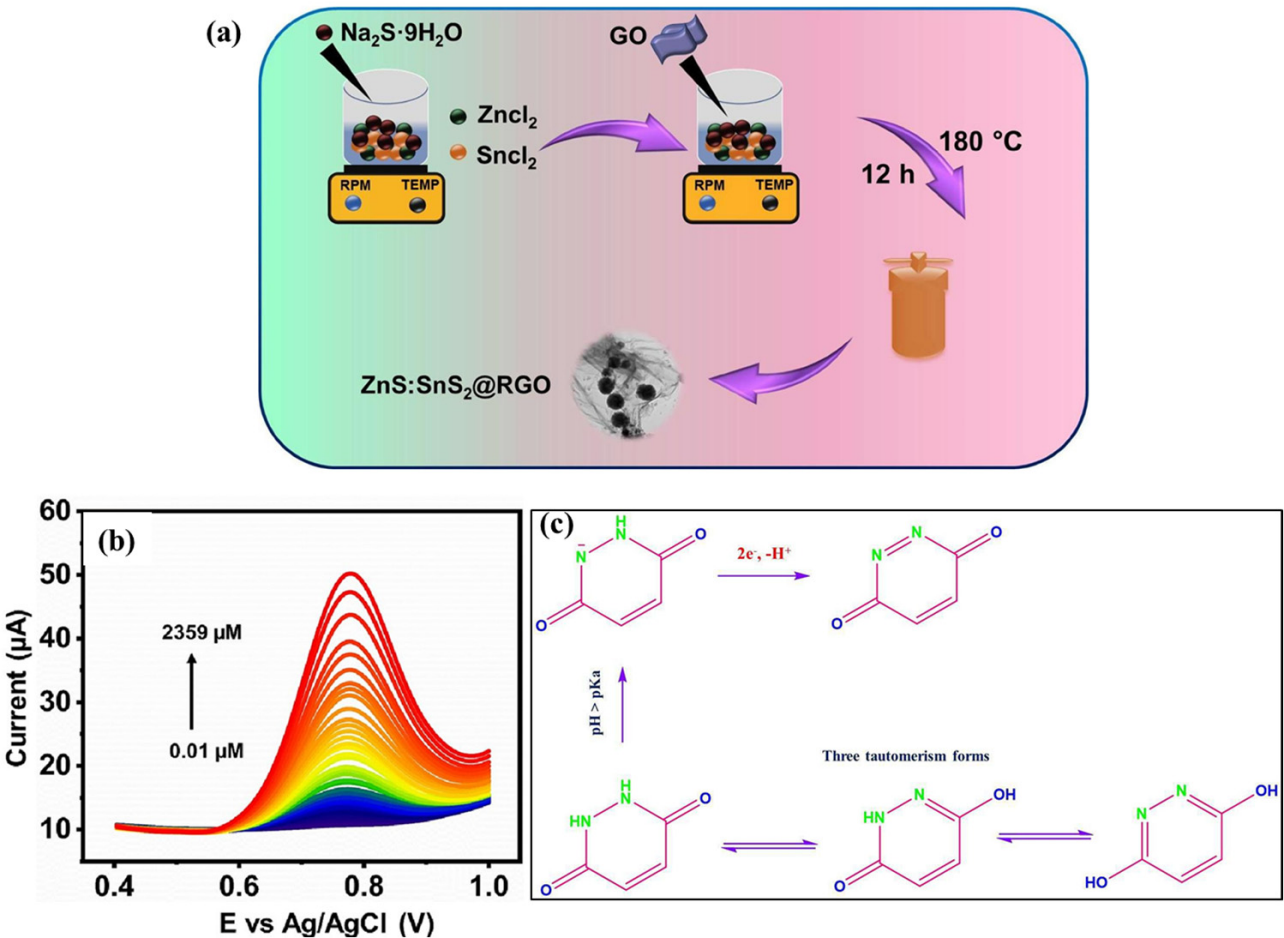
(**a**) Schematic graph shows the formation of ZnS/SnS2/RGO. (**b**) DPV curves of the ZnS/SnS_2_/RGO in various concentrations of MD. (**c**) Electrochemical oxidation process for MD detection. Reproduced with permission [63].

The authors used polyvinyl alcohol (PVA) and nafion to prepare the electrode material and explored it for the determination of GSH. It was also found that the proposed electrode modifier exhibits high porosity, high electrical conductivity, and large surface area, which may improve electron transport, whereas the presence of O and S vacancies enhances the electro-oxidation of GSH. The reaction mechanism for GSH detection is depicted in Figure 2d. It was also observed that the ZnS/MnO_2_-MOF exhibits high electrocatalytic activity for GSH detection and limit of detection, i.e., LOD of 6.88 nM was achieved with sensitivity of 8.45 µA nM^−1^ cm^−2^. The proposed electrochemical sensor was also selective for GSH detection in the presence of various interfering substances, such as UA, Cl^−^, Na^+^, Ca^2+^, bovine serum albumin (BSA), K^+^, ascorbic acid (AA), and glucose (Glu). MXene materials are promising conductive materials to enhance the electrical conductivity of less conductive and semiconducting materials. The incorporation of MXene such as metal carbides and metal nitride with metal sulfides or metal oxides not only improves conductive properties but also enhances catalytic properties for electrochemical reactions. In this connection, Arif et al. [54] reported the formation of a niobium carbide (Nb_2_C) MXene-ZnS composite to improve the electrical conductivity and electrochemical performance of ZnS for DA detection. The authors used differential pulse voltammetry for the determination of DA and observed that the current response increases with increasing DA concentration. The LOD of 1.39 μM with a linear range of 0.09 to 0.82 mM and sensitivity of 12.1 μA μM^−1^ was obtained for DA detection under optimized conditions. The authors also reported excellent selectivity for DA detection in the presence of AA, Glu, and citric acid (CA). In another report [55], it was proposed that the early-stage detection of microRNA (miRNA) is of great significance for the diagnosis of tumors. A layer-by-layer (LBL) assembled ZnS/MXene/CdS (ZMC) heterostructure was developed for the detection of miRNA. The electrode was also modified with HS-H1 DNA through disulfide bonds. The optimized electrode exhibited an LOD of 0.36 fM with acceptable recovery of 98.27% to 101.35% in human serum samples. Kim et al. [56] explored the conductive and catalytic properties of gold (Au) to improve the electrochemical sensing performance of a ZnS-based para-nitrophenol (p-NP) sensor. The Au-modified ZnS (AZS) was coated on a glassy carbon electrode, i.e., GCE and its electrochemical characteristics were examined using cyclic voltammetry (CV technique). The AZS-modified GCE shows an LOD of 320 nM with a linear range of 150 to 2000 nM and reasonable selectivity for p-NP detection. Real sample recovery also revealed the potential of the AZS-modified GCE as a promising electrode for p-NP detection. In a previous report [57], a ZnS film was also fabricated on an Au electrode for electrochemical quartz crystal microbalance (EQCM). The reports show that ZnS is a promising electrode material if combined with supporting conductive materials. Therefore, it is clear that the fabrication of ZnS with conductive substrates and materials is of significance for the construction of highly sensitive electrochemical sensors. In recent years, it was also reported that the simultaneous determination of targeted analytes has several advantages such as cost-effectiveness and energy/time savings. It is well known that levodopa (LD) and UA are two important neurotransmitters, which are generally associated with several neurological diseases. Thus, the determination of LD and UA would be of great significance to overcome the negative impacts of the imbalance of LD and UA on human health. In this regard, three-dimensional (3D) graphene foam (GF) was obtained using the chemical vapor deposition method [58]. The ZnS NPs were in situ grown on to the surface of 3D GF via the hydrothermal method. The obtained Zn NPs/3D GF were employed as sensing material for the monitoring of the LD and UA using the DPV technique. The LOD of 0.043 μM, linear range of 0.5 to 60 μM and sensitivity of 2.34 μA·μM^−1^·cm^−2^ were obtained for LD detection, whereas an LOD of 0.042 μM, linear range of 0.5 to 60 μM and sensitivity of 2.39 μA·μM^−1^·cm^−2^ were achieved for UA detection, indicating that the proposed ZnS NPs/3D GF are sensitive for the determination of LD and UA. Alkahtani et al. [59] adopted the one-pot sonochemical approach for the preparation of the ZnS NPs/reduced graphene oxide (rGO) nanosheets. The surface morphological features of the ZnS NPs/rGO composite were studied by using scanning electron microscopy and transmission electron microscopy (SEM and TEM). The obtained ZnS NPs/rGO composite-modified electrode was explored for the determination of daclatasvir (DAC) and hydroxychloroquine (HCQ). The electrochemical activity of the ZnS NPs/rGO-modified electrode was improved due to the existence of synergism between ZnS NPs and rGO. The LODs of 0.456 and 0.498 nM were observed for HCQ and DAC detection, respectively. The ZnS NPs/rGO/GCE enabled the determination of HCQ and DAC in biological samples with acceptable recoveries, which suggested its potential for practical applications. In another study [60], a ZnS@Ni_2_P/rGO composite was prepared and electrodeposited onto a GCE surface for chlorpyrifos (CPS) detection in farmland recirculated water. The SEM and XRD studies revealed that the rippled rGO nanosheets may facilitate porous/crystalline growth of ZnS@Ni_2_P, which may further improve the surface area and electron transfer. The electrochemical studies showed high sensitivity of 0.89799 μA/μM with a linear range of 25 to 375 μM and LOD of 0.004 μM. The proposed sensor also exhibited reliable performance in real water samples with acceptable recoveries, suggesting its potential for CPS monitoring in agricultural wastewater. In a previous report [61], a ZnS NP-decorated composite graphene paper electrode (CGPE) was explored as a sensing material for the construction of DA sensor. This proposed DA sensor showed an LOD of 0.0042 µM and linear range of 0.1 to 2300 µM and excellent stability, with decent reproducibility and durability. It is well known that maleic hydrazide (MD) is widely used to prevent sprouting in vegetables and stored root crops. However, its serious genotoxic and carcinogenic risks motivated the researchers to develop the electrochemical sensor for the monitoring of MD in food samples. In this connection, a novel ZnS/SnS2/RGO heterojunction was fabricated using the hydrothermal method, as shown in Figure 3a [62]. The hydrothermal reaction was carried out at 180 °C for 12 h, which yielded ZnS/SnS2/RGO. The obtained ZnS/SnS_2_/RGO was coated on the GCE surface, and its electrochemical activity for MD detection was examined by employing CV and DPV. The ZnS/SnS_2_/RGO-modified GCE exhibited excellent electrocatalytic properties for MD detection and current response linear increases with increasing concentration of MD, as shown in Figure 3b. The synergistic effects between the ZnS/SnS_2_ and RGO improve the detection of MD, and the fabricated sensor delivered an LOD of 0.02 µM with a linear range of 0.01 to 2359 µM. The electrochemical oxidation of MD at the ZnS/SnS_2_/RGO-modified GCE surface is described in Figure 3c.

**Figure 4:**
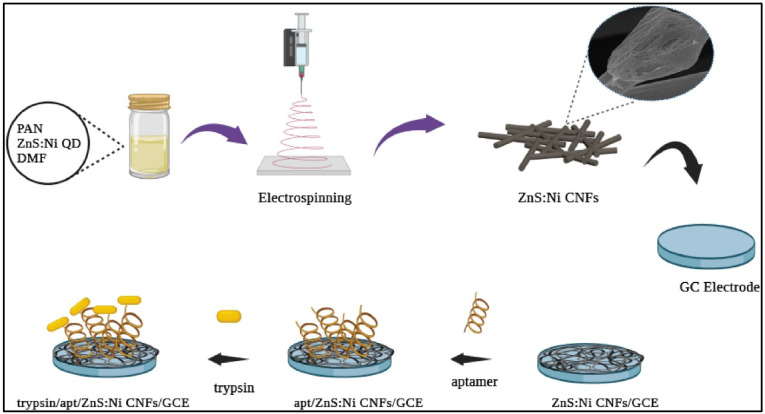
Schematic demonstration of the electrode fabrication process. Reproduced with permission [71].

A ZnS/graphene composite was also explored for the construction of a biosensor towards the determination of SARS-CoV-2 genes with decent sensitivity [63]. It is suggested that ZnS can be used for the fabrication of biosensors for clinical diagnostic applications. Zhao et al. [64] proposed the fabrication of a ZnS/RGO composite for the construction of a UA sensor. The authors observed that the prepared ZnS has different morphological features under different conditions used for the synthesis of ZnS. The ZnS nanoflakes/RGO material-based biosensor showed sensitivity of 534.5 μA·cm^−2^·mM^−1^ and a linear range of 0.01 to 2 mM with an LOD of 0.048 μM. The presence of a flake-like structure of ZnS and the conductivity of RGO were responsible for this improved electrochemical performance from the proposed UA sensor. The CdS@ZnS core–shell quantum dots (QDs) were electrochemically prepared and employed for the fabrication of an electrochemical sensor towards the determination of propranolol (PRO) [65]. This proposed electrochemical sensor was designed and constructed by modifying the active surface area of the GCE with an MWCNT film-coated CdS@ZnS QDs (GCE/MWCNTs/CdS@ZnS). The authors used square wave voltammetry (SWV) for the determination of propranolol (PRO) using GCE/MWCNTs/CdS@ZnS. This sensor exhibited a linear range of 0.06 to 27 μM with an LOD of 12 nM for the determination of PRO. Naik et al. [66] designed and prepared a ZnS/Au/f-MWCNT composite through the pulsed laser-assisted method followed by a wet chemical process. The proposed electrode was used as an electrocatalyst for 4-nitrophenol (4-NP) detection, and the obtained results suggested that an optimized electrode can deliver an LOD of 30 nM, linear range of 10 to 150 µM with sensitivity of 0.8084 μA μM^−1^ cm^−2^ under the optimized conditions. This improved performance may be ascribed to the presence of abundant active sites and enhanced electron transfer. Previously [67], Mn-doped ZnS QDs were synthesized through the hydrothermal method and characterized by various sophisticated techniques. The L-cysteine-capped Mn-ZnS QDs were also integrated with the MWCNTs and deposited on GCE to form the ZnS/MnQDs-MWCNTs/GCE. This sensor exhibited a linear range of 90 to 1200 nM, LOD of 28 nM, and sensitivity of 0.001 μA nM^−1^, with excellent selectivity for hydrazine detection. It is understood that nitrite plays a vital role in nitrification and denitrification within the nitrogen cycle, but its negative effects motivated the researchers to develop the nitrite sensor [68]. In this connection, the authors combined ZnS with carboxylated CNTs and phase-transition BSA (PTB) to develop the electrochemical sensor. The ZnS-CNT/PTB-based sensor delivered an LOD of 0.73 nM and linear range of 10 nM to 0.4 mM. The Mn^2+^-doped ZnS nanostructures were also prepared through the reflux method. Mn^2+^-doped ZnS was deposited on the GCE surface and explored for the determination of isoprenaline (ISP) using the DPV technique [69]. The proposed sensor exhibited sensitivity of 2.1 μA μM^−1^, a linear range of 0.5 to 30 μM and LOD of 90 nM for ISP detection. In another study [70], a hydrogen peroxide (H_2_O_2_) electrochemical sensor was developed by using a CdS/ZnS composite as the electrocatalyst. The authors adopted the hydrothermal method for the preparation of the electrocatalyst. It was found that ZnS consisted of a cubic structure with a nanorod shape, whereas CdS comprised a hexagonal structure with spherical-shaped architecture. The authors found that the proposed electrochemical sensor may detect H_2_O_2_ with an LOD of 0.35 µM and sensitivity of 430 µA mM^−1^ cm^−2^. The presence of a wide linear range of 1 to 1870 µM and excellent selectivity for H_2_O_2_ detection suggests its potential for practical applications. The ZnS/Ni QDS-embedded carbon nanofibers (ZnS/Ni/CNFs) were also synthesized through the electrospinning method followed by a carbonization process, which was used as a substrate for the construction of an aptamer-based trypsin sensor, as shown in Figure 4 [71].

The fabricated electrochemical aptasensor exhibited a wide detection range of 0.1 fg/mL to 600 ng/mL and an LOD of 0.03 fg/mL, with excellent reproducibility and selectivity for trypsin detection. The real sample studies using a human serum sample exhibited satisfactory results for trypsin detection, which suggested its potential applications in healthcare systems. The Sr-modified ZnS QDs (Sr@ZnS QDs) were synthesized through the thermal decomposition method [72]. The obtained Sr@ZnS QDs were explored for the electrochemical detection of H_2_O_2_. The presence of active sites, Sr^2+^/Zn^2+^ interactions with H_2_O_2_, and synergism in the prepared electrocatalyst improved the electrochemical activity of the Sr@ZnS QD-modified screen-printed carbon electrode (SPCE) for H_2_O_2_ detection. Thus, sensitivity of 12.68 μA·mM^−1^·cm^−2^, LOD of 75.9 μM and linear range of 1 to 80 mM were observed for H_2_O_2_ detection. It is understood that morphine is a potent analgesic, but an overdose may cause severe health risks. Thus, the detection of morphine is of great significance. In this regard, a low-cost electrochemical sensor was developed by employing ZnS/MIL-125 nanocomposite-modified GCE [73]. The CV and DPV analysis-based studies revealed that the proposed electrochemical sensor is sensitive for the determination of morphine with an LOD of 3 nM and linear range of 0.01 to 75 μM. Additionally, the proposed sensor was found to be highly selective, reproducible, and demonstrated satisfactory results in real samples. In another previous study [74], a signal-on solid-state ECL sensor was developed for paclitaxel (PTX) detection using luminol@CNTs and CdTe-ZnS@HAP. It was observed that luminol acts as the luminophore, CdTe-ZnS acts as a co-reaction accelerator, whereas CNTs and HAP act as carriers to enhance the active sites. Therefore, a linear range of 3 × 10^−12^ to 3 × 10^−7^ M and LOD of 1 × 10^−12^ M were obtained for PTX detection. The authors also found that the proposed sensor is efficient for the detection of PTX analysis in serum samples. The ZnS/graphitic carbon nitride (ZnS@CNS) binary nanosheet composite was also prepared using the sonochemical method [75]. The ZnS@CNS-modified GCE showed higher electrocatalytic activity for flutamide detection using CV, DPV, and amperometry techniques. It was observed that the presence of the synergistic effects of ZnS and CNS may provide abundant active sites and fast electron transport. Thus, the proposed sensor may exhibit a broader linear range and decent LOD for flutamide detection. This suggests that the ZnS-based composite is a promising electrocatalyst for flutamide detection. In another previous effort [76], a novel carbon paste electrode (CPE) modified with PVA-capped Mn-doped ZnS NPs/g-C_3_N_4_ (PVA-Mn/ZnS/g-C_3_N_4_@CPE) was fabricated for the determination of metoclopramide hydrochloride (MCH). The authors adopted CV and SWV techniques for the determination of MCH and reported an LOD of 5 nM, with satisfactory recovery of MCH in urine and water samples. Ciprofloxacin (CIP) is a widely used fluoroquinolone antibiotic, which may contaminate the environment due to improper disposal [77]. Thus, fabrication of a CIP electrochemical sensor is of great significance. Therefore, a ZnS–PANI composite-modified electrode was explored for CIP sensing applications. The optimized conditions revealed that the proposed sensor is highly sensitive and stable for CIP detection, and an LOD of 0.5 μM can be obtained. The proposed CIP sensor also showed excellent selectivity for CIP detection in the presence of various interfering antibiotics, such as ENR, OFL, and LEV. Gokulkumar et al. [78] proposed the fabrication of a cost-effective electrochemical sensor for the detection of 3-nitrophenol (3-NP). In this context, GCE was modified with the ZnS NPs/PDA-coated functionalized CNF. The presence of the synergistic effect between ZnS and PDA@f-CNF may provide a larger surface area, decent conductivity, adsorption capacity, and improved electrocatalytic activity. Thus, the fabricated 3-NP sensor demonstrated decent linearity, good LOD, repeatability, reproducibility, and stability, with satisfactory detection of 3-NP in urine samples, suggesting its potential for real-time sensing applications. It is understood that salmonella is a major foodborne pathogen, which is associated with poultry [79]. The rapid detection of salmonella in chicken products is of great importance for food safety. Thus, a 3D-printed electrochemical immunosensor for salmonella typhimurium detection was developed using sandwich immunoassay/biotinylated antibodies and Cd/Se ZnS QDs. The proposed sensor exhibited decent performance for the determination of salmonella typhimurium. In another study [80], a novel electrochemical immunosensor was fabricated for tuberculosis (TB) detection using CdSe/ZnS QDs/functionalized silica nanoparticle (CdSe/ZnS QD/SiNP/SPCE)-modified SPCE. This fabricated sensor detects the CFP10–ESAT6 antigen through enzyme catalase-mediated antigen–antibody interaction using the DPV technique. The LOD of 1.5 × 10^−10^ g/mL and excellent reproducibility were observed under the optimized conditions. These observations suggest that the ZnS is a promising electrocatalyst for electrochemical sensing applications. The electrochemical sensing parameters, such as LOD, sensitivity, linear range, etc., are summarized in Table 2.

## 4. ZnS-Based Materials in SCs

**Figure 5:**
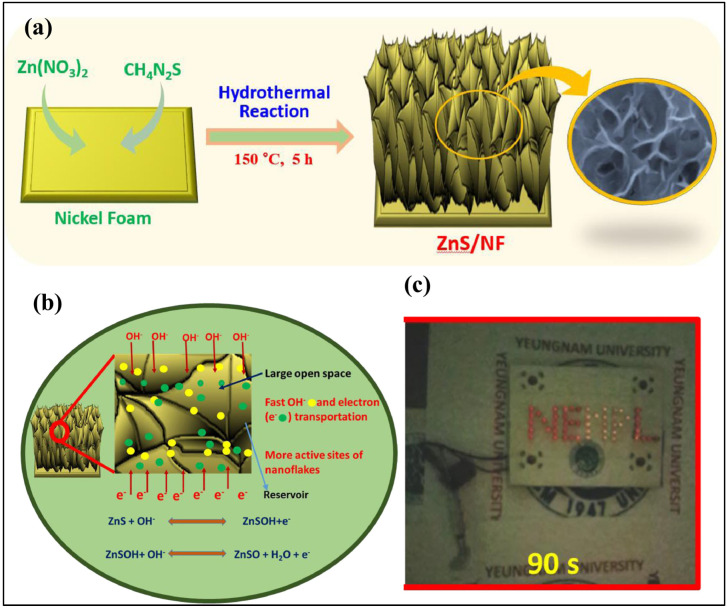
(**a**) Schematic graph shows the formation of ZnS/NF. (**b**) Mechanism involved for electrochemical reactions. (**c**) LED light. Reproduced with permission [83].

It is understood from Section 3 that ZnS-based hybrid materials exhibit decent electrochemical properties for the construction of electrochemical sensors/biosensors. It was also found that the ZnS-based materials were also used for SC applications. In this section, we summarize the recent progress in ZnS-based materials for SC applications. The ZnS was deposited on the nickel foam (NF) electrode by employing a simple approach [81]. The fabricated ZnS/NF electrode exhibited reasonable electrochemical activity and energy storage properties. The presence of ZnS on NF improves the charge transport properties of the ZnS/NF electrode, and specific capacitance of 1827.5 F/g was achieved at a current density of 15 A/g with stability of 3000 cycles. This report shows that ZnS may be utilized as an electrode modifier and energy storage material for the development of next-generation supercapacitor applications. The hydrothermal method was also adopted for the fabrication of ZnS on NF [82]. The jute-derived activated carbon was used as a negative electrode, whereas ZnS/NF was adopted as a positive electrode. The specific capacitance of 781 F/g at 0.5 A/g was obtained using ZnS/NF as a positive electrode. The proposed SCs also showed excellent stability of 10,000 cycles, revealing the presence of energy storage properties in ZnS. In another study [83], thin ZnS nanoflakes were also fabricated on NF through a benign hydrothermal method, as shown in Figure 5a. The authors achieved specific capacity of 659 C/g at 2 A/g, with reasonable stability. The mechanism for the proposed SCs is described in Figure 5b. The fabricated SCs also displayed stability of 2000 cycles, and two series-connected devices powered 52 red LEDs for about 90 s, as shown in Figure 5c.

**Figure 6:**
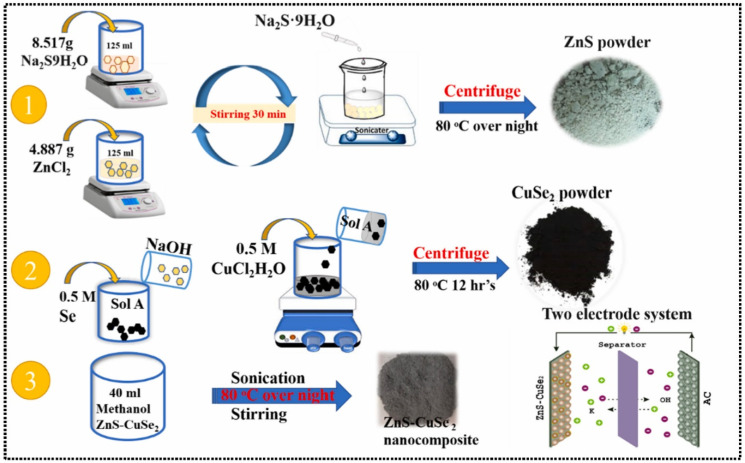
Schematic representation of the preparation of ZnS, CuSe_2_, and ZnS–CuSe_2_. Reproduced with permission [99].

The ZnS nanosheets were successfully obtained through a simple hydrothermal method, and their electrochemical properties for supercapacitor applications were tested in a 3 M potassium hydroxide (KOH) electrolyte [84]. The proposed electrode material displayed a decent specific capacitance of 536 F/g at 1 A/g, with excellent coulombic efficiency of 96% and stability of 5000 cycles. Previously [85], a rapid, one-step, and low-cost microwave-assisted method was explored for the preparation of mesoporous ZnS nanosheets, which exhibited larger surface area of 120 m^2^/g, with pore diameter of more than 22 nm. This may provide abundant electrochemical active sites and short ion/electron diffusion paths. Therefore, a ZnS nanosheet-based modified electrode showed high specific capacitance of 2282 F/g at 1 A/g in a 2 M KOH electrolyte. The fabricated SC device also exhibited reasonable specific capacitance of 252.5 F/g, with stability of 10,000 cycles. Nanda et al. [86] proposed the formation of ZnS/MnO_2_ MOF hydrogel using novel strategies. The XRD-based studies revealed that the prepared ZnS/MnO_2_ MOF has a cubic structure, whereas morphological studies indicated the uniform dispersion of the prepared material with a porous structure. Electrochemical studies for supercapacitor applications were carried out in the presence of a PVA-KOH electrolyte system. The proposed electrode material exhibited specific capacitance of 112 F/g at 1.5 A/g, with energy density of 303 Wh/kg and power density of 1050 W/kg. The proposed electrode also maintained device performance up to 63% over 2000 cycles, which may be ascribed to the high conductivity of the proposed electrode material and solid electrolyte. Godlaveeti et al. [87] also reported the hydrothermal synthesis of a ZnS/MnO_2_ composite for hybrid SC applications. The authors found that the ZnS/MnO_2_-based electrode has better electrochemical performance compared to the pristine MnO_2_ or ZnS-based electrode. This may be ascribed to the presence of synergism between ZnS and MnO_2_. Therefore, a specific capacitance of 254.3 F/g was obtained for the ZnS/MnO_2_-based electrode. This suggested that the incorporation of ZnS with metal oxides improved the electrochemical performance of the ZnS for SC applications. Thus, in this connection, Hadi et al. [88] also explored the potential of ZnS for SC applications by incorporating it with iron oxide (Fe_2_O_3_). The authors observed that the prepared Fe_2_O_3_/ZnS composite has a specific surface area of 40.44 m^2^/g, which is higher compared to the ZnS (30.71 m^2^/g) and Fe_2_O_3_ (20.48 m^2^/g). The CV, electrochemical impedance spectroscopy (EIS), and galvanostatic charge discharge (GCD) techniques were used to study the electrochemical properties of the Fe2O3/ZnS composite for SC applications. The EIS studies showed that the Fe_2_O_3_/ZnS composite has high electrical conductivity and demonstrated stability of 5000 cycles. The specific capacitance of 1095 F/g at 1 A/g was also obtained for the Fe_2_O_3_/ZnS composite-based electrode. The improved electrochemical performance of the Fe_2_O_3_/ZnS composite-based electrode may be attributed to the high specific surface area, more active sites and synergism in the prepared Fe_2_O_3_/ZnS composite. In another previous study [89], copper oxide (CuO) was also combined with ZnS to form the ZnS/CuO composite for asymmetric SC applications. The prepared material exhibited the presence of a microsphere-shaped surface morphology, with enhanced surface area and charge storage properties. The fabricated electrode showed specific capacitance of 561 F/g and stability of 5000 cycles. It is clear that the combined effects of the ZnS/CuO composite enhance the charge storage properties of the proposed SCs. Abdullah et al. [90] reported the hydrothermal synthesis of MnO_2_, ZnS, and ZnS/MnO_2_ composites for SC applications. The structural and chemical properties of the prepared materials were characterized by using various techniques such as XRD and SEM, etc., whereas electrochemical performance was tested in a 2 M KOH electrolyte system. The ZnS/MnO_2_-based electrode exhibited specific capacitance of 1002 F/g at 1 A/g, which may be ascribed to the synergism of the electrode material. Zinc oxide (ZnO) has decent catalytic properties and has been explored in electrochemical applications. TheZnO@ZnS composite was synthesized using the hydrothermal method [91]. The ZnO@ZnS composite-based electrode was utilized as an SC electrode, which exhibited specific capacitance of 440.6 F/g, with stability of 5500 cycles. The excellent catalytic properties of the ZnO@ZnS composite enhance the supercapacitive energy storage properties of the fabricated electrode. In another study [92], a novel ternary ZnO-ZnS-CdS heterostructure was prepared under simple conditions. It was observed that urchin-like ZnO was coated with ZnS and CdS NPs, which enhanced the surface functionality of the fabricated electrode for electrochemical reactions. The fabricated electrode showed improved capacity of 434 mAh/g compared to the ZnO/ZnS-based electrode. Ghotbi et al. [93] utilized layered nanoreactors for the fabrication of a novel metal oxide/metal sulfide/heteroatom-doped carbon nanostructure. In this connection, Zn-based layered hydroxide (zinc hydroxide sulfate) was modified with gallate anions and heat-treated under inert gas to form a ZnO/ZnS/S-doped carbon composite. Furthermore, acid etching yielded ZnS/S-doped carbon and 3D S-doped carbon frameworks. The ZnS/S-doped carbon electrode demonstrated high specific capacitance of 1047 F/g under the optimized conditions. Riaz et al. [94] proposed the formation of a novel urchin-like W_18_O_49_ and ball-like ZnS nanospheres using the hydrothermal method. The specific capacitance of 517 F/g at 1 A/g was achieved for the fabricated electrode in a 3 M KOH electrolyte. The W_18_O_49_-ZnS || MnO_2_-KOH system exhibited stability of 10,000 cycles. It is understood that metal selenides such as tungsten selenide (WSe_2_) are promising electrode materials for electrochemical applications. It would be a great idea to combine the ZnS with metal selenides to form the hybrid composites for SC applications. In this regard, nickel cobalt phosphate (NiCoP) coated with a WSe_2_@ZnS composite was synthesized through the hydrothermal method [95]. The NiCoP/WSe_2_@ZnS hybrid composite-based electrode exhibited specific capacity of 2416 F/g at 1 A/g with stability of 5000 cycles. The authors also observed that the fabricated asymmetric SC device may deliver specific capacity of 178 F/g at 0.5 mA/g. In another study [96], cobalt selenide (CoSe_2_) and ZnS-based composites were prepared using the wet chemical method. The synthesized material displayed excellent electrochemical properties for SC applications. The structural and morphological studies revealed that ZnS forms cubic microspheres, whereas CoSe_2_ shows a snowflake-like surface structure with smooth surfaces. The presence of synergism between the CoSe_2_ and ZnS enhanced the energy storage properties of the resulting composite. Ahmad et al. [97] adopted the solvothermal method for the preparation of a ZnS/iron selenide (FeSe_2_) composite. The authors found that ZnS and FeSe_2_-based electrodes exhibit specific capacitance of 266.2 F/g and 294.3 F/g, respectively. In contrast, the proposed composite material-based electrode exhibited improved specific capacitance of 444.4 F/g under the optimized conditions, with cyclic stability of 12,000. Kumar et al. [98] designed and fabricated a honeycomb-like WSe_2_@ZnS-coated NH_4_NiPO_4_.H_2_O hybrid using benign synthetic protocols. It was observed that the obtained WSe_2_@ZnS-coated NH_4_NiPO_4_.H_2_O has decent electrical conductivity and electrochemical energy storage properties, which makes it an promising electrode material for SCs. The higher specific capacitance of 1542 F/g was obtained at a current density of 1 A/g, with enhanced stability. The asymmetric SC device also demonstrated specific capacitance of 146 F/g at 1 A/g, with excellent stability of 10,000 cycles (retained 92% of initial capacitance). Another novel composite of ZnS with copper selenide (CuSe_2_) was also fabricated through the sonochemical-assisted synthesis method, as shown in Figure 6 [99]. It was observed that the prepared ZnS has a cubic structure, whereas CuSe_2_ possesses an orthorhombic structure, with decent phase purity and crystallinity. The electrochemical studies also indicated the presence of improved electrocatalytic and energy storage properties, which make it a promising electrode material for SC applications. The authors achieved specific capacitance of 95 F/g at 5 A/g for the proposed asymmetric SCs, with stability of 8000 cycles (retention of initial capacitance = 81.8%).

**Figure 7:**
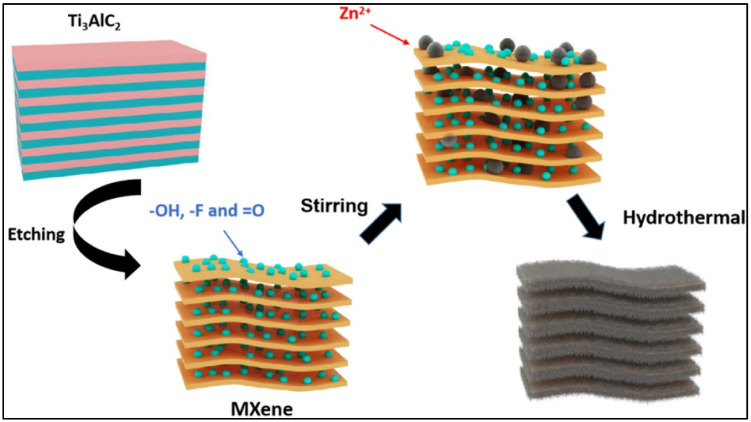
Schematic illustration of the fabrication of MXene/ZnS composite. Reproduced with permission [115].

The ZnS/cobalt sulfide (Co_3_S_4_) nanoscrubber composite was also prepared using the hydrothermal method [100]. The GCD and CV techniques were employed to investigate the electrochemical properties of the proposed electrode materials for SC applications. The obtained results exhibited specific capacitance of 593 F/g at 0.5 A/g with reasonable stability of 2000 cycles. The in situ hydrothermal method was explored for the preparation of the MoS_2_/ZnS composite, and the zinc level was varied in the range of 5 mol% to 20 mol% [101]. The XRD results confirmed the formation of the prepared composite with decent phase purity. The MoS_2_/ZnS composite-based electrode exhibited specific capacitance of 564 C/g under the optimized conditions. The specific capacitance of the MoS_2_/ZnS composite-based electrode was higher compared to the pure MoS_2_-based electrode. This improved performance of the MoS_2_/ZnS composite-based electrode may be attributed to the presence of synergism in the MoS_2_/ZnS composite. In another previous investigation [102], the MoS_2_/ZnS composite was also utilized as a binder-free electrode material for SC applications. The improved specific capacitance of 1628.3 F/g was obtained for the MoS_2_/ZnS composite-modified electrode at 1 A/g, indicating that the MoS_2_/ZnS composite is a promising material for SC applications. Azizi et al. [103] developed activated carbon from rice husk (ACRH) combined with a binary ZnS/FeS composite. The ZnS/FeS/ACRH composite was obtained through the hydrothermal method. The specific capacitance of 1207 C/g with stability of 5000 cycles was obtained under the optimized conditions. The authors also found that fabricated SCs powered blue and red LEDs for about 40 s which suggested their potential for industrial applications. Ma et al. [104] also reported the hydrothermal synthesis of ZnS/MoS_2_ composite films on Mo foil. The prepared ZnS/MoS_2_ composite film showed high specific capacitance of 956.3 F g^−1^ at 10 mA/cm^−2^, which was higher than that of the pristine MoS_2_-based electrode (150 F/g). The improved electrochemical performance may be attributed to the robust nanoparticle structure, strong substrate/material contact, low resistance, and synergism between ZnS and MoS_2_. Jia et al. [105] fabricated a NiCo-OHS@ZnS electrode for SC applications. The synthesized NiCo hydroxide exhibited a hierarchical hollow-sphere structure. The optimized conditions revealed that a specific capacitance of 1655 C/g at 1 A/g can be achieved using the proposed electrode material. Au NP-coated nickel sulfide (Ni_3_S_2_)/ZnS/carbon-TiN nanotube arrays (Au@Ni_3_S_2_/ZnS/C-TiN NTAs) were developed using an electrodeposition-assisted approach [106]. The presence of active sites, sulfur vacancies, improved OH- adsorption and improved ion/electron transport enhanced the electrochemical performance of the fabricated Au@Ni_3_S_2_/ZnS/C-TiN NTAs. The authors developed asymmetric SCs using Au@Ni_3_S_2_-ZnS/C-TiN NTAs and Au-coated ZnS/NF as negative and positive electrodes, respectively, which demonstrated decent cyclic stability and high energy density. In another report [107], the ZnS/MoS_2_ heterostructure was also developed on NF through a homemade chemical vapor deposition (CVD) method. The authors observed that interconnected nanofibers and nanorods may improve the surface area, porosity, active sites, and chemical stability of the prepared ZnS/MoS_2_. The ZnS/MoS_2_/NF electrode displayed specific capacitance of 3540 F/g at 1 A/g with stability of 20,000 cycles, which is higher than the MoS_2_/NF electrode (1666 F/g). In another research article [108], a nanosphere-decorated flower-like ZnS/zinc cobalt sulfide (ZnCo_2_S_4_)@Ni(OH)_2_ heterostructure was also developed through the solvothermal method. The synthesized ZnS/ZnCo_2_S_4_@Ni(OH)_2_ electrode exhibited high specific capacitance of 2276.9 F/g at 1 A/g with stability of 5000 cycles. Tian et al. [109] reported that S vacancy-rich sheet-like Ni_3_S_2_@ZnS composites can be grown on 3D porous NF electrodes using a two-step electro-deposition method. The synthesized Ni_3_S_2_@ZnS/NF electrode exhibited specific capacitance of 1141.9 C/g at 19 A/g. It was also found that the proposed electrode material retained 86.7% capacitance after 50,000 cycles. The S vacancies improved surface activity and hydroxide diffusion through modulating local electron density, accelerating interfacial redox reactions and enhanced electrochemical performance. The synthesized phosphorus (P)-doped Ni_2_S_3_/Co_3_S_4_/ZnS nanowire/nanosheet arrays were prepared through hydrothermal/annealing treatments [110]. The proposed electrode material may provide active sites for electrochemical reactions and facilitates ion transport. The P doping also enhanced electrical conductivity. Therefore, the fabricated electrode exhibits specific capacity of 2716 F/g at 1 A/g with the retention of 89% capacitance after 9000 cycles. Khan et al. [111] reported the preparation of a ZnS/CdS composite on an NF electrode through the hydrothermal method. It was observed that the prepared electrode material exhibits snow-like nanospheres, which may improve the electron transport. The fabricated electrode demonstrated specific capacitance of 739 F/g at 1 A/g, with 92.8% retention of initial capacitance after 6500 cycles. The intrinsic conducting polymers (ICPs) like polypyrrole (PPy), which is well known for its high conductivity, redox activity, and charge storage, were explored for SC applications [112]. The PPy/ZnS composite was obtained through an in situ oxidative polymerization approach. The authors observed that the proposed electrode material may deliver specific capacitance of 452.22 F/g at 100 mV/s. This reasonable capacitance may be ascribed to the presence of synergistic interactions and higher electrical conductivity of the PPy/ZnS composite. In another study [113], a ZnS QD-decorated polyaniline (PANI) composite was also fabricated through the co-precipitation-assisted chemical oxidation polymerization approach. The electrochemical performance of the PANI/ZnS QDs composite was evaluated in 1 M sulfuric acid (H_2_SO_4_) using CV, GCD, and EIS methods. The GCD studies revealed that the proposed electrode may deliver specific capacitances of 893.75 F/g at 0.5 A/g, with stability of 1000 cycles. Gao et al. [114] developed a micro-3D electrode (where template-shaped ZnS nanorods were anchored on hollow carbon nanofibers followed by directional growth of ZnCo-LDH nanosheets, and coated with MXene layer) via electrostatic spraying. This prepared hierarchical structure composite forms a stable polymetallic array with a conductive network and enables fast ion/electron transfer. The synthesized HZCNF@ZS-LDH@MX electrode exhibited decent specific capacitance of 830 F/g, which may be ascribed to the high specific surface area of the electrode material (740 m^2^/g). The proposed electrode also demonstrated excellent cycling stability of 20,000 cycles. Zhang et al. [115] also proposed that two-dimensional titanium carbide MXene (Ti_3_C_2_) is a promising electrode material for SCs. In addition, MXene can also be used to improve the conductivity and catalytic properties of the low or semi-conducting material. The MXene/ZnS composite was synthesized through the solvothermal method, as displayed in Figure 7. The resulting composite exhibited specific capacity of 2347.2 C/g at 1 A/g under the optimized conditions.

The 3D sponge-like reduced graphene oxide (rGO)/CNT-decorated ZnS composite was synthesized via the microwave-assisted hydrothermal method [116]. The obtained rGO/MWCNTf-ZnS composite displayed improved electrical conductivity and electrochemical properties for SCs. However, specific capacity of 95 F/g was observed, which needs to be improved by introducing novel strategies. Rani et al. [117] reported the electrochemical performance of the ZnS/NiS and MWCNT/ZnS/NiS composites synthesized via a simple hydrothermal method for SC applications. The MWCNT/ZnS/NiS composite-based electrode exhibited specific capacitance of 2267 F/g at 1 A/g, which is higher than the ZnS/NiS (1693 F/g), ZnS (853 F/g), and NiS (1127 F/g)-based electrodes. This may be attributed to the presence of synergistic interactions, improved conductivity and active sites. In another study [118], Co(OH)_2_ nanowires were grown on ZnS nanosheets through a multi-step hydrothermal method. It was observed that the presence of nanowires improved charge transfer and increased active sites. The ZnS@Co(OH)_2_-based electrode exhibited specific capacitance of 1063 F/g at 1 A/g with stability of 6000 cycles. The binder-free Zn_1−x=y=z_Co_x_Cu_y_Fe_z_S (ZCCFS) was synthesized using the hydrothermal method [119]. The ZCCFS (Z5) electrodes exhibited high areal capacitance of 3.561 F/cm^2^ with stability of 10,000 cycles. Another report also explored the potential of activated carbon and ZnS for SC applications [120]. The ZnS NP-based SCs demonstrated specific capacitance of 371.5 F/g at 1 A/g under the optimized conditions. In another research article [121], the AC/ZnS-Ni_7_S_6_/Ni(OH)_2_ nanoflower composite was fabricated on an NF electrode through a three-step hydrothermal method. The prepared material displayed an ultrathin nanoflower structure, which may offer abundant active sites and decent conductivity. The prepared material exhibited specific capacity of 1034.52 C/g at 1 A/g, with stability of 3000 cycles. Similarly, Shohag et al. [122] explored the potential of ZnS for SC applications. The authors prepared an L-glutamic acid/ZnS (L-GA/ZnS) composite via the benign solvent casting method. The prepared composite-based electrode exhibited decent energy storage performance. These reports indicated that ZnS-based materials are promising materials for SC applications. The specific capacitances of the reported ZnS-based SCs are summarized in Table 3.

## 5. Conclusions and Future Perspectives

It is worth mentioning that ZnS is a promising electrode modifier for electrochemical applications, such as sensors and energy storage applications. ZnS has good catalytic properties for redox reactions but has limited conductivity. Thus, various reports proposed that a doping strategy may improve the conductivity of the pristine ZnS. The incorporation of conductive materials such as graphene, MXene and polymers may also provide the synergistic interactions and enhance the conductivity of the ZnS-based hybrid composites. The ZnS and its composite-based electrode materials exhibited excellent electrochemical performance for sensors and SC applications. However, some challenges remain, which need to be overcome.

1. The long-term stability of ZnS-based materials is limited for practical applications.
2. The exact mechanism for the SCs and sensors needs to be studied in detail.
3. The ZnS/MXene may be a suitable material, but preparation of MXene requires harsh conditions. Thus, environmentally friendly methods should be developed.
4. DPV-based studies may suffers from low selectivity due to the presence of similar isomers or interfering substances.
5. The long-term cyclic stability of the SCs is still limited.
6. ZnS can be integrated with high surface area and conducting materials for SC applications.
7. Future research may explore electrochemical sensors for portable, miniaturized devices, flexible and wearable or smartphone-controlled devices for environmental monitoring and healthcare systems.

## Figures and Tables

**Table 1:** Advantages/disadvantages of various synthesis methods.

Synthesis Method	Advantages	Disadvantages
Hydrothermal	High yield, pH, temperature, time, and morphology of the materials can be controlled	Time consuming, limited scalability, energy consumption, and high pressure requirements
Microwave	Rapid reaction rate, uniform heating, high yield, and control over temperature, pressure and time	High cost for equipment, scalability issues, safety concerns, and non-uniform heating in bulk synthesis
Sol-gel	Purity, homogeneity and control over precursor concentration	Cracking of films, long processing time, moisture sensitivity, and low scalability
Electrochemical	Reaction parameters, including current density, voltage, and time can be controlled	Limited to conductive substrates only such as FTO, complex optimization, limited control over crystal growth and low yield
Ultrasonication	Simple, eco-friendly, uniform particle formation, and decent reaction yield	Relies of radiation sources on ultrasonic radiation, limited control over particle morphology

**Table 2:** Electrochemical sensing performance of the reported ZnS-based sensors.

Electrode Material	Analyte	Detection Technique	LOD (µM)	Linear Range (µM)	Sensitivity	Real Sample	Refs.
ZnS nanoflakes	UA	Amp	1.51	0.01–2.0 mM	34.28 µA·cm^−2^·mM^−1^	-	[52]
ZnS nanoparticles	UA	Amp	1.79	0.01–1.5 mM	43.18 µA·cm^−2^·mM^−1^	-	[52]
ZnS urchin-like nanostructures	UA	Amp	0.7	0.01–1.7 mM	76.12 µA·cm^−2^·mM^−1^	-	[52]
ZnS/MnO_2_/MOF HG	GSH	DPV	6.88 nM	10 nM to 10 mM	8.45 µA·nM^−1^ cm^−2^	Stimulated blood serum	[53]
GCE/Nb_2_C/ZnS	DA	DPV	1.39	0.09–0.82 mM	12.1 μA/μM	-	[54]
ZnS	p-NP	SWV	0.68	-	5 nA/nM	-	[56]
ZnS NPs/3D GF	UA	DPV	0.042	0.5–60	2.39 μA·μM^−1^·cm^−2^	Human plasma	[58]
ZnS NPs/3D GF	LD	DPV	0.043	0.5–60	2.34 μA·μM^−1^·cm^−2^	Human plasma	[58]
ZnS NPs@rGO/GCE	DAC	DPV	0.498 nM	7–65 nM	-	Urine and plasma	[59]
ZnS NPs@rGO/GCE	HCQ	DPV	0.456 nM	5–65 nM	-	Urine and plasma	[59]
ZnS/SnS_2_/rGO	MD	DPV	0.02	0.01–2359	0.207 µA µM^−1^ cm^−2^	Water and potato	[62]
Activated GCE/MWCNTs/CdS@ZnS	PRO	SWV	12 nM	0.06–27	-	Urine, human blood plasma, and tablet	[65]
ZnS/Mn QDs/MWCNTs/GCE	Hydrazine	DPV	28 nM	90–1200 nM	0.001 µA nM^−1^	Tap, mineral and lake water	[67]
PTB-ZnS/CNTs/SPE	NO_2_^−^	i-t	0.00073	0.00001–0.4 mM	-	Soil sample	[68]
Mn.ZnS(II)	ISP	DPV	90 nM	0.5–30	66.88 μA μM^−1^ cm^−2^	-	[69]
Sr@ZnS QDs	H_2_O_2_	DPV	75.9	1–80 mM	12.68 μA⋅mM^−1^⋅cm^−2^	-	[71]
ZnS@C_3_N_4_	flutamide	LSV	12.6 nM	0.05–1320.15	2.352 μA μM^−1^ cm^−2^	Urine and serum	[75]
PVA-Mn:ZnS/g-C_3_N_4_@CPE	MCH	DPV	5.03 nM	20 nM–10 µM	-	Pharmaceutical tablets	[76]
ZnS/f-CNF@PDA/GCE	3-NP	DPV	0.85	0.05–497	0.12 μA μM^−1^ cm^−2^	Urine	[78]

**Table 3:** Electrochemical performance of the reported ZnS-based SCs.

Electrode Material	Specific Capacitance (F/g)	Current Density (A/g)	Electrolyte	Cyclic Stability	Retention (%)	Refs.
ZnS/NF	1827.5	15	1 M KOH	3000	68.42	[81]
ZnS nanoflakes	659 C/g	2	3 M KOH	5000	87	[83]
ZnS nanosheets	2282	1	2 M KOH	10,000	95	[85]
PVA-ZnS/MnO_2_	112	1.4	PVA-KOH gel	20,000	63	[86]
ZnS/MnO_2_	254.3	0.5	3 M KOH	5000	82	[87]
Fe_2_O_3/_ZnS	1095	1	3 M KOH	5000	-	[88]
ZnS/MnO_2_	1002	1	1 M KOH	5000	95	[90]
NiCoP/WSe_2_@ZnS	2416	1	2 M KOH	5000	96	[95]
ZnS@FeSe_2_ (AZ-2//AC)	110	1	3 M KOH	12,000	88.1	[97]
NH_4_NiPO_4_.H_2_O/WSe_2_@ZnS	1542	1	3 M KOH	10,000	97	[98]
ZnS/Co_3_S_4_	593	0.5	2 M KOH	2000	-	[100]
MoS_2_/ZnS||MoS_2_/ZnS	145 C/g	5 mV/s	2 M KOH	3000	90	[101]
MoS_2_/ZnS	1628.3	1	1M H_2_SO_4_	1000	78.3	[102]
ZnS/MoS_2_	956.3	10 mA/cm^3^	1 M KCl	2000	85.7	[104]
ZnS/MoS_2_	3032	1	2 M KOH	20,000	97	[107]
ZnS/ZnCo_2_S_4_@Ni(OH)_2_	2276.9	1	6 M KOH	10,000	94.5	[108]
Ni_3_S_2_ @ZnS/NF	1903	19	1 M KOH	50,000	86.7	[109]
P-Ni_2_S_3_/Co_3_S_4_/ZnS	2716	1	3 M KOH	9000	89	[110]
PANI/ZnS QDs	358	0.5	1 M H_2_SO_4_	1000	97.8	[113]
MXene/ZnS	2347.2 C/g	1	2 M KOH	5000	89.88	[115]
MWCNTs/ZnS/NiS	2267	2	2 M KOH	5000	93	[117]
Zn_1−*x*=*y*=*z*_Co*_x_*Cu*_y_*Fe*_z_*S	117.5 mF cm^−2^	2 mA cm^−2^	2 M KOH	10,000	72	[119]
Activated carbon/ZnS	371.5	1	3 M KOH	10,000	82.5	[120]
AC/ZnS-Ni_7_S_6_/Ni(OH)_2_	1034.52	1	2 M KOH	3000	93.8	[121]

## References

[1] Fatima S., Hossain M.E., Alnour M., Kanwal S., Rehman M.Z., Esquivias M.A. (2024). Assessing the damage to environmental pollution: Discerning the impact of environmental technology, energy efficiency, green energy and natural resources. Sustainability.

[2] Gajdzik B., Wolniak R., Nagaj R., Žuromskaitė-Nagaj B., Grebski W.W. (2024). The influence of the global energy crisis on energy efficiency: A comprehensive analysis. Energies.

[3] Tal A. (2025). The environmental impacts of overpopulation. Encyclopedia.

[4] Nsair R., Alzubi A.B. (2025). Globalization, financial risk, and environmental degradation in China: The role of human capital and renewable energy use. Sustainability.

[5] Munzhelele E.P., Mudzielwana R., Ayinde W.B., Gitari W.M. (2024). Pharmaceutical contaminants in wastewater and receiving water bodies of South Africa: A review of sources, pathways, occurrence, effects, and geographical distribution. Water.

[6] Estrada-Almeida A.G., Castrejón-Godínez M.L., Mussali-Galante P., Tovar-Sánchez E., Rodríguez A. (2024). Pharmaceutical pollutants: Ecotoxicological impacts and the use of agro-industrial waste for their removal from aquatic environments. J. Xenobiot..

[7] Aib H., Parvez M.S., Czédli H.M. (2025). Pharmaceuticals and microplastics in aquatic environments: A comprehensive review of pathways and distribution, toxicological and ecological effects. Int. J. Environ. Res. Public Health.

[8] Babuji P., Thirumalaisamy S., Duraisamy K., Periyasamy G. (2023). Human health risks due to exposure to water pollution: A review. Water.

[9] Piscopo M., Marinaro C., Lettieri G. (2024). The multifaceted impact of environmental pollutants on health and ecosystems. Biomolecules.

[10] Noh D., Oh E. (2024). Estimation of environmental effects and response time in gas-phase explosives detection using photoluminescence quenching method. Polymers.

[11] Hameedat F., Hawamdeh S., Alnabulsi S., Zayed A. (2022). High performance liquid chromatography (HPLC) with fluorescence detection for quantification of steroids in clinical, pharmaceutical, and environmental samples: A review. Molecules.

[12] Chun S., Muthu M., Gopal J. (2022). Mass spectrometry as an analytical tool for detection of microplastics in the environment. Chemosensors.

[13] Li X., Jin Y., Zhu N., Yin J., Jin L.Y. (2024). Recent developments of fluorescence sensors constructed from pillar[n]arene-based supramolecular architectures containing metal coordination sites. Sensors.

[14] Ojeda C.B., Rojas F.S. (2006). Recent development in optical chemical sensors coupling with flow injection analysis. Sensors.

[15] Elmorsi E.T., Lai E.P.C. (2025). Optimization of capillary electrophoresis by central composite design for separation of pharmaceutical contaminants in water quality testing. Environments.

[16] Baranwal J., Barse B., Gatto G., Broncova G., Kumar A. (2022). Electrochemical sensors and their applications: A review. Chemosensors.

[17] Ahmad K., Karmegam D., Oh T.H. (2025). Progress in Bi_2_WO_6_-based materials for electrochemical sensing and supercapacitor applications. Molecules.

[18] Zhu H., Li M., Cheng C., Han Y., Fu S., Li R., Cao G., Liu M., Cui C., Liu J. (2023). Recent advances in and applications of electrochemical sensors based on covalent organic frameworks for food safety analysis. Foods.

[19] Panneer Selvam S., Chinnadayyala S.R., Cho S., Yun K. (2020). Differential pulse voltammetric electrochemical sensor for the detection of etidronic acid in pharmaceutical samples by using rGO-Ag@SiO_2_/Au PCB. Nanomaterials.

[20] Pogăcean F., Măgeruşan L., Turza A., Pruneanu S. (2025). Graphene-modified electrode for linear sweep voltammetric sensing of catechol. Chemosensors.

[21] Kavieva L., Ziyatdinova G. (2022). Voltammetric sensor based on SeO_2_ nanoparticles and surfactants for indigo carmine determination. Sensors.

[22] Lazaro A., Villarino R., Girbau D., Haji-Hashemi H., Prieto-Simon B. (2025). Low-cost wireless device for DNA sensing using square wave voltammetry. Chemosensors.

[23] Liu N., Zhao G., Liu G. (2020). Coupling square wave anodic stripping voltammetry with support vector regression to detect the concentration of lead in soil under the interference of copper accurately. Sensors.

[24] Liu C., Chen D., Zhu C., Liu X., Wang Y., Lu Y., Zheng D., Fu B. (2023). Fabrication of a disposable amperometric sensor for the determination of nitrite in food. Micromachines.

[25] Farina R., Scalese S., Corso D., Capuano G.E., Screpis G.A., Coniglio M.A., Condorelli G.G., Libertino S. (2024). Chronoamperometric ammonium ion detection in water via conductive polymers and gold nanoparticles. Molecules.

[26] Wang X.L., Jin E.M., Sahoo G., Jeong S.M. (2023). High-entropy metal oxide (NiMnCrCoFe)_3_O_4_ anode materials with controlled morphology for high-performance lithium-ion batteries. Batteries.

[27] Saleem S., Khalid S., Malik M.A., Nazir A. (2024). Review and outlook of zinc sulfide nanostructures for supercapacitors. Energy Fuels.

[28] Subasinghage K., Gunawardane K. (2024). Supercapacitor-assisted energy harvesting systems. Energies.

[29] Czagany M., Hompoth S., Keshri A.K., Pandit N., Galambos I., Gacsi Z., Baumli P. (2024). Supercapacitors: An efficient way for energy storage application. Materials.

[30] Saba S., Alsharari A.M., Aldaleeli N.Y., Aljohani M.M., Hamdalla T.A. (2025). Structural, electrochemical, and supercapacitor characterization of double metal oxides doped within ZIF-8 composites. Processes.

[31] Tadesse M.G., Ahmmed A.S., Lübben J.F. (2024). Review on conductive polymer composites for supercapacitor applications. J. Compos. Sci..

[32] Nandeesh K.N., Prashantha K. (2025). Recent advances on metal sulfides as next-generation electrodes for supercapacitor energy storage: A holistic review. Next Mater..

[33] Olabi A.G., Abbas Q., Abdelkareem M.A., Alami A.H., Mirzaeian M., Sayed E.T. (2023). Carbon-based materials for supercapacitors: Recent progress, challenges and barriers. Batteries.

[34] Otgonbayar Z., Yang S., Kim I.-J., Oh W.-C. (2023). Recent advances in two-dimensional MXene for supercapacitor applications: Progress, challenges, and perspectives. Nanomaterials.

[35] Zhao J., Yang L., Li R., Zhou Y. (2023). One-step synthesis of Fe-based metal–organic framework (MOF) nanosheet array as efficient cathode for hybrid supercapacitors. Inorganics.

[36] Chakrabarti A., Alessandri E. (2024). Syntheses, properties, and applications of ZnS-based nanomaterials. Appl. Nano.

[37] Alhassan S., Alshammari A.H., Alotibi S., Alshammari K., Mohamed W.S., Hadia N.M.A. (2024). Structural and optical properties of nickel-doped zinc sulfide. Nanomaterials.

[38] Mane S.M., Wagh K.S., Teli A.M., Beknalkar S.A., Shin J.C., Lee J. (2024). One-pot facile synthesis of a cluster of ZnS low-dimensional nanoparticles for high-performance supercapacitor electrodes. Micromachines.

[39] Alam M.W., Al Qahtani H.S., Albalawi H., Aamir M., Bilal M., Mir T.A., Souayeh B., Zaidi N. (2022). Enhanced electrodes for supercapacitor applications prepared by hydrothermal-assisted nanosheet-shaped MgCo_2_O_4_@ZnS. Crystals.

[40] Xu S.-D., Wu L.-C., Adil M., Sheng L.-F., Zhao Z.-Y., Xu K., Chen X. (2025). Developing a CeS_2_/ZnS quantum dot composite nanomaterial as a high-performance cathode material for supercapacitor. Batteries.

[41] Ibupoto Z.H., Khun K., Liu X., Willander M. (2013). Hydrothermal synthesis of nanoclusters of ZnS comprised on nanowires. Nanomaterials.

[42] Gao S., Lu Y., Ma T., Liu H., Zhang J. (2025). Preparation and photocatalytic hydrogen production of pink ZnS. Inorganics.

[43] Bailón-Ruiz S.J., Cedeño-Mattei Y., Núñez-Colón A.M., Torres-Torres K. (2024). Fast one-step microwave-assisted synthesis of iron-doped ZnS for photocatalytic applications. Crystals.

[44] Wang W., Lee G.-J., Wang P., Qiao Z., Liu N., Wu J.J. (2020). Microwave synthesis of metal-doped ZnS photocatalysts and applications on degrading 4-chlorophenol using heterogeneous photocatalytic ozonation process. Sep. Purif. Technol..

[45] Stanić V., Etsell T.H., Pierre A.C., Mikula R.J. (1997). Sol–gel processing of ZnS. Mater. Lett..

[46] Yang K., Guo Q., Li H., Hao X., Ma Y., Yang M., Zhai T., Savilov S.V., Lunin V.V., Xia H. (2018). Highly efficient sol–gel synthesis for ZnS@N,S co-doped carbon nanosheets with embedded heterostructure for sodium ion batteries. J. Power Sources.

[47] Hamla M., Benaicha M., Chetbani Y., Dilmi O. (2024). Electrochemical synthesis and characterization of zinc sulfide (ZnS) semiconducting thin films from citrate-based plating bath. J. Appl. Electrochem..

[48] Samskruthi K.P., Ananda S., Alnaggar G. (2021). Electrochemical synthesis of ZnS/In_2_S_3_ nanocomposite for photocatalytic oxidation of acetic acid and its substituents: Efficiency compared with ZnS, In_2_S_3_ and TiO_2_. Chem. Data Collect..

[49] Xu J., Ji W., Lin J., Tang S., Du Y. (1998). Preparation of ZnS nanoparticles by ultrasonic radiation method. Appl. Phys. A.

[50] Annalakshmi M., Kumaravel S., Chen S.-M., Balasubramanian P., Balamurugan T.S.T. (2020). A straightforward ultrasonic-assisted synthesis of zinc sulfide for supersensitive detection of carcinogenic nitrite ions in water samples. Sens. Actuators B Chem..

[51] Elnouby M.S., El-Shazly O., El-Wahidy E.F., Ramadan M., Farag A.A.M., Roushdy N. (2023). Optimized synthesis, structural and electrical enhancement of zinc sulfide nanoparticles for electrochemical sensor applications. Optik.

[52] Zhao Y., Wei X., Peng N., Wang J., Jiang Z. (2017). Study of ZnS nanostructures-based electrochemical and photoelectrochemical biosensors for uric acid detection. Sensors.

[53] Singh P., Mukundan G., Badhulika S. (2024). ZnS/MnO_2_ metal–organic framework-based conductive hydrogel for highly selective and sensitive detection of glutathione in serum samples. Microchem. J..

[54] Arif N., Gul S., Sohail M., Rizwan S., Iqbal M. (2021). Synthesis and characterization of layered Nb_2_C MXene/ZnS nanocomposites for highly selective electrochemical sensing of dopamine. Ceram. Int..

[55] Wang T., Jiang D., Shi Y., Wang Z., Liu G., Lin S., Zhou H., Wang Q. (2024). ZnS/MXene/CdS heterostructure modified electrode for ultrasensitive miRNA-21 assay with signal amplified photoelectrochemical strategy. Sens. Actuators B Chem..

[56] Kim Y., Giribabu K., Kim J.G., Lee J.B., Hong W.G., Huh Y.S., Kim H.J. (2019). Electrochemical sensors based on Au–ZnS hybrid nanorods with Au-mediated efficient electron relay. ACS Sustain. Chem. Eng..

[57] Krinitsyn D.O., Romanchenko A.S., Vorob’ev S.A., Likhatskii M.N., Karacharov A.A., Krylov A.S., Volochaev M.N., Mikhlin Y.L. (2021). Synthesizing zinc sulfide films on the gold surface as the sensor for electrochemical quartz crystal microbalance. Russ. J. Electrochem..

[58] Yue H.Y., Wu P.F., Huang S., Gao X., Song S.S., Wang W.Q., Guo X.R. (2019). Simultaneous electrochemical determination of levodopa and uric acid based on ZnS nanoparticles/3D graphene foam electrode. Microchem. J..

[59] Alkahtani S.A., Mahmoud A.M., Mahnashi M.H., AlQarni A.O., Alqahtani Y.S.A., El-Wekil M.M. (2021). Facile one-pot sonochemical synthesis of layered nanostructure of ZnS NPs/rGO nanosheets for simultaneous analysis of daclatasvir and hydroxychloroquine. Microchem. J..

[60] Zheng W., Sui J., Chen T., Qi Q., Lv Y. (2021). Synthesis and application of nanocomposite of ZnS, Ni_2_P and reduced graphene oxide as electrochemical sensor for determination of chlorpyrifos in farmland water. Int. J. Electrochem. Sci..

[61] Erçarıkcı E., Aksu Z., Topçu E., Dağcı Kıranşan K. (2022). ZnS nanoparticles-decorated composite graphene paper: A novel flexible electrochemical sensor for detection of dopamine. Electroanalysis.

[62] Disouza F.P.D., Ruspika S., Chen S.-M., Balaji R., Meena Devi J., Peng J.-Y., Jothi A.I. (2023). Synergetic effect of ZnS:SnS_2_/reduced graphene oxide heterostructures for electrochemical detection of carcinogenic pollutant maleic hydrazide. Microchem. J..

[63] Sarwar S., Lin M.C., Amezaga C., Wei Z., Iyayi E., Polk H., Wang R., Wang H., Zhang X. (2023). Ultrasensitive electrochemical biosensors based on zinc sulfide/graphene hybrid for rapid detection of SARS-CoV-2. Adv. Compos. Hybrid Mater..

[64] Zhao Y., Peng N., Gao W., Hu F., Zhang C., Wei X. (2024). ZnS and reduced graphene oxide nanocomposite-based non-enzymatic biosensor for the photoelectrochemical detection of uric acid. Biosensors.

[65] Arab N., Fotouhi L., Salis A. (2021). Electrosynthesised CdS@ZnS quantum dots decorated multiwalled carbon nanotubes for analysis of propranolol in biological fluids and pharmaceutical samples. Microchem. J..

[66] Naik S.S., Lee S.J., Theerthagiri J., Yu Y., Choi M.Y. (2021). Rapid and highly selective electrochemical sensor based on ZnS/Au-decorated functionalized multiwalled carbon nanotube nanocomposites produced via pulsed laser technique for detection of toxic nitro compounds. J. Hazard. Mater..

[67] Amini N., Gholivand M.B., Shamsipur M., Maleki A., Naderi K., Marzban N., Lee S.-M., Jeon C. (2022). Modification of glassy carbon surface using L-cysteine-capped Mn-doped ZnS quantum dots and multiwall carbon nanotube nanocomposite: Application to determine hydrazine in water samples. Desalin. Water Treat..

[68] Wang Z., Wan Y., Zhang Y., Zhang B., Li M., Jin X., Yang T., Meng G. (2024). 3D porous conductive matrix based on phase-transited BSA and covalent coupling-stabilized transition ZnS–CNT for antifouling and on-site detection of nitrite in soil. J. Hazard. Mater..

[69] Sabbaghan M., Ghalkhani M., Hosseini M., Ghanbari M. (2021). Mn-doped ZnS synthesis in DABCO-based ionic liquid: Morphology and electrochemical sensing performance for isoprenaline analysis. J. Ind. Eng. Chem..

[70] Othmani A., Maddouri A., Derbali M., Touati F., Dhaouadi H. (2022). A 1D/0D CdS@ZnS nanocomposite as an electrochemical sensor for hydrogen peroxide detection. J. Mater. Sci. Mater. Electron..

[71] Shantiaei H., Roushani M., Mohammadi F. (2025). Electrospinning ZnS:Ni quantum dots into carbon nanofibrous structure as a base for the electrochemical aptasensor for detection of trypsin. Sens. Actuators Rep..

[72] Tithito T., Traiwatcharanon P., Kondee S., Seekaew Y., Chaloeipote G., Seesaard T., Reunchan P., Pon-On W., Wongchoosuk C. (2025). Thermal decomposition synthesis of Sr-modified ZnS quantum dots for hydrogen peroxide electrochemical sensing. Electrochim. Acta.

[73] Akrami Z., Sohouli E. (2025). Preparation of an ultra-sensitive electrochemical sensor for morphine measurement using the ZnS/MIL-125 nanocomposite. J. Alloys Compd..

[74] Li H., Tian L., Yang S., Li C., Song Y., Li R., Guo Y., Lu J. (2024). A signal-on electrochemiluminescence sensor based on luminol@carbon nanotubes and CdTe–ZnS@hydroxyapatite for the detection of paclitaxel. Microchem. J..

[75] Chen P.-Y., Reddy T.K., Rajaji U., Alothman A.A., Govindasamy M. (2024). Optimization of electrochemical sensitivity in anticancer drug quantification through ZnS@CNS nanosheets: Synthesis via accelerated sonochemical methodology. Ultrason. Sonochem..

[76] Patil Y.N., Megalamani M.B., Nandibewoor S.T. (2023). Graphitic carbon nitride infused with PVA–Mn:ZnS modified carbon sensor for electrochemical investigation of metoclopramide hydrochloride. Diam. Relat. Mater..

[77] Ali S.K., Alamier W.M., Hasan N., Ahmed S., Ansari A., Imran M. (2023). Synergistic nanomaterials: Zinc sulfide–polyaniline for ciprofloxacin electrochemical sensing. Appl. Phys. A.

[78] Gokulkumar K., Priscillal I.J.D., Wang S.-F. (2022). Deep eutectic solvent-mediated synthesis of PDA-coated f-CNF doped ZnS nanoparticles for electrode modification: Innovative sensing platform for determination of pollutant 3-nitrophenol. J. Alloys Compd..

[79] Angelopoulou M., Kourti D., Mertiri M., Petrou P., Kakabakos S., Kokkinos C. (2023). A 3D-printed electrochemical immunosensor employing Cd/Se ZnS QDs as labels for the rapid and ultrasensitive detection of Salmonella typhimurium in poultry samples. Chemosensors.

[80] Mohd Bakhori N., Yusof N.A., Abdullah J., Wasoh H., Ab Rahman S.K., Abd Rahman S.F. (2020). Surface-enhanced CdSe/ZnS QD/SiNP electrochemical immunosensor for the detection of Mycobacterium tuberculosis by combination of CFP10–ESAT6 for better diagnostic specificity. Materials.

[81] Tian F., Liu S., Li H., Li D., Han X. (2022). Preparation and electrochemical capacitance of different micromorphology zinc sulfide on nickel foam for asymmetric supercapacitor. J. Energy Storage.

[82] Rauf M., Shah S.S., Shah S.N.A., Haq T.U., Shah J., Ullah A., Ahmad T., Khan Y., Aziz M.A., Hayat K. (2022). Facile hydrothermal synthesis of zinc sulfide nanowires for high-performance asymmetric supercapacitor. J. Saudi Chem. Soc..

[83] Hussain I., Mohapatra D., Dhakal G., Lamiel C., Sayed M.S., Sahoo S., Mohamed S.G., Kim J.S., Lee Y.R., Shim J.-J. (2021). Uniform growth of ZnS nanoflakes for high-performance supercapacitor applications. J. Energy Storage.

[84] Ajith Kumar K., Gnanavel B., Mohan K.S., Manoranjitham R., Backiyalakshmi N., Nallusamy S., Rao Bhaviripudi V.V., Kavinkumar T., Thirumurugan A. (2025). Hydrothermal fabrication of zinc sulfide nanosheets for high-performance supercapacitors. Mater. Lett..

[85] Hassan N.U., Jabeen N., Younas W., Ahmed F., Hussain A., Asif S.U., Alghamdi M.M., Naveed M. (2024). Efficient hybrid supercapacitor performance enabled by large surface area of 2D mesoporous zinc sulfide nanosheets synthesized via microwaves. J. Electroanal. Chem..

[86] Nanda O.P., Singh P., Banothu Y.N., Kumar R., Badhulika S. (2024). Hydro/solvothermally grown ZnS/MnO_2_-metal organic framework based hydrogel for all-solid-state flexible supercapacitor. J. Energy Storage.

[87] Godlaveeti S.K., Alshgari R.A., Mushab M., Mingqiang L., Ying H. (2025). ZnS/MnO_2_ nanocomposite electrodes: A dual approach for superior supercapacitor and safety open structure lithium-ion battery. J. Mol. Struct..

[88] Hadi M. (2025). Development of novel and excellent Fe_2_O_3_/ZnS nanohybrid electrode for supercapacitor. Inorg. Chem. Commun..

[89] Khan A.U., Tahir K., Almarhoon Z.M., Albaqawi H.S., Alanazi A.A., Al-Shehri H.S., Althagafi T.M., Elboughdiri N., Al-Saeedi S.I., Zaki M.E.A. (2024). A new binder-free ZnS–CuO microsphere: A battery-type electrode material for asymmetric supercapacitor. J. Energy Storage.

[90] Abdullah M., John P., Jabbour K., Ahmad M.I., Khan S., Waheed M.S., Albaqami M.D., Sheikh M., Ehsan M.F., Ashiq M.N. (2024). Improvement in capacitive performance of ZnS with MnO_2_ via composite (ZnS/MnO_2_) strategy developed by hydrothermal technique. J. Energy Storage.

[91] Shah M.Z.U., Sajjad M., Hou H., BiBi S., Shah A. (2022). Hydrothermal synthesis of ZnO@ZnS heterostructure on Ni foam: A binder-free electrode for high power and stable hybrid supercapacitors. Mater. Lett..

[92] BiBi S., Shah M.Z.U., Sajjad M., Shafi H.Z., Amin B., Bajaber M.A., Shah A. (2022). A new ZnO–ZnS–CdS heterostructure on Ni substrate: A binder-free electrode for advanced asymmetric supercapacitors with improved performance. Electrochim. Acta.

[93] Ghotbi M.Y., Sikiru S., Rajabi A., Soleimani H., Kou L., Ansari M.N.M., Ramachandaramurthy V.K. (2024). ZnO/ZnS/carbon nanocomposite-derived sulfur-doped carbon nanosheets using a layered nanoreactor: Towards advanced supercapacitor electrodes and devices. Chem. Eng. J..

[94] Riaz J., Cao J., Bibi A., Arif M., Muhammad D. (2024). Hydrothermal synthesis of ball-like ZnS nanospheres decorated urchin-like W_18_O4_9_ nanospheres as electrode for high power and stable hybrid supercapacitor. Mater. Lett..

[95] Ajith Kumar K., Gnanavel B., Joshua J.R., BaQais A., Sharmila V., Alam M.W. (2023). Decoration of sea-urchin-like NiCoP coated with WSe_2_@ZnS as an advanced electrode material for hybrid supercapacitors. Electrochim. Acta.

[96] Ahmad S.A., Shah M.Z.U., Rahman S.U., Arif M., Lu J., Huang T., Ahmad A., Al-Kahtani A.A., Tighezza A.M., Sajjad M. (2023). CoSe_2_@ZnS microsphere arrays with remarkable electrochemical performance for hybrid asymmetric supercapacitor. J. Energy Storage.

[97] Ahmad S.A., Shah M.Z.U., Rahman S.U., Arif M., Javed M.S., Sufyan M., Huang T., Al-Saeedi S.I., Song P., Sajjad M. (2022). Facile synthesis of hierarchical ZnS@FeSe_2_ nanostructures as new energy-efficient cathode material for advanced asymmetric supercapacitors. J. Sci. Adv. Mater. Devices.

[98] Ajith Kumar K., Gnanavel B., Alam M.W., Joshua J.R., Sharmila V., BaQais A. (2024). Design of honeycomb-like WSe_2_@ZnS coated NH_4_NiPO_4_·H_2_O hybrids for flexible all-solid-state asymmetric supercapacitors. J. Energy Storage.

[99] Ahmad S.A., Shah M.Z.U., Ullah S.M., Rahman S.U., Ullah M.S., Eldin S.M., Song P., Sajjad M. (2023). High power aqueous hybrid asymmetric supercapacitor based on zero-dimensional ZnS nanoparticles with two-dimensional nanoflakes CuSe_2_ nanostructures. Ceram. Int..

[100] Dhilip Kumar R., Sreevani K., Shanmugavalli V., Nagarani S., Dhinakaran V., Balamurali R. (2022). ZnS–Co_3_S_4_ nanoscrubber synthesized by hydrothermal route for pseudo-supercapacitors. Mater. Lett..

[101] Ismail K.B.M., Kumar M.A., Prasath J., Mahalingam S., Jayavel R., Arivanandhan M., Kim J. (2025). Enhanced supercapattery performance of hydrothermally synthesized MoS_2_/ZnS nanocomposites. J. Energy Storage.

[102] Yadav A., Sharma A.K., Dubey A., Dey K.K., Chaudhary D.K., Dhawan P.K. (2025). Enhanced pseudocapacitive performance of MoS_2_/ZnS nanocomposites for advanced supercapacitor applications. Mater. Lett..

[103] Azizi S., Askari M.B., Salarizadeh P. (2025). ZnS/FeS/activated carbon derived from rice husk loaded on nickel foam as a novel electrode material for supercapacitors. Diam. Relat. Mater..

[104] Ma Y., Jia Y., Lin Y., Shi W. (2021). ZnS/MoS_2_ film grown on Mo foil as binder free electrode for supercapacitor. Chem. Phys..

[105] Jia S., Zhao Q., He M., Zhang T. (2024). Hierarchical construction of amorphous Ni–Co–OHS@ZnS hollow spheres with optimized reaction kinetics via ion exchange-etching strategy for energy storage. Chem. Eng. J..

[106] Tian F., Wang H., Li H., Bai X., Wu J., Geng F., Hu J., Ren L., Zhu T., Wei T. (2024). Enhancement of the electrochemical performance of Ni_3_S_2_–ZnS/carbon-coated TiN nanotube arrays through Au-ion beam sputtering. J. Energy Storage.

[107] Raza A., Rasheed A., Farid A., Yousaf M., Ayub N., Khan I.A. (2024). Synthesis of binder-free nanofibers ZnS/MoS_2_/NiF electrode material for asymmetric supercapacitor applications. J. Energy Storage.

[108] Qian J., Xu J., Lu Z., Wang Y. (2023). Preparation of nanosphere-interspersed flower-like heterostructure of ZnS/ZnCo_2_S_4_@Ni(OH)_2_ and its application for asymmetric solid-state supercapacitor. J. Energy Storage.

[109] Tian F., Wu J., Hu J., Lu Q., Guo S., Ren L., Han W., Zhu T., Wei T. (2023). Controlled synthesis of sulfur-vacancy-enriched sheet-like Ni_3_S_2_@ZnS composites for asymmetric supercapacitors with ultralong cycle life. J. Alloys Compd..

[110] Gao Y., Yue X., Dong Y., Zheng Q., Lin D. (2024). High-efficiency activated phosphorus-doped Ni_2_S_3_/Co_3_S_4_/ZnS nanowire/nanosheet arrays for energy storage of supercapacitors. J. Colloid Interface Sci..

[111] Khan M.U., Khan A.U., Al-Saeedi S.I., Tahir K., Alanazi A.A., Almarhoon Z.M., Althagafi T.M., Zaki M.E.A., Hassan H.M.A., Wu M. (2024). Hydrothermally synthesized binder-less ZnS/CdS on nickel foam as promising hybrid supercapacitor electrode materials. J. Electroanal. Chem..

[112] Ansari S., Kumar S., Nayak D., Mandal G., Bauri J., Choudhary R.B. (2024). Zinc sulfide (ZnS) incorporated polypyrrole (PPy) matrix for high-performance supercapacitor. Mater. Today Proc..

[113] Salah N., Shehab M., El Nady J., Ebrahim S., El-Maghraby E.M., Sakr A.-H. (2023). Polyaniline/ZnS quantum dots nanocomposite as supercapacitor electrode. Electrochim. Acta.

[114] Gao Y., Huang Y., Zong M., Jiang T., Zhang S., Zhang Z. (2025). Designing micro-3D hierarchical HZCNF@ZS-LDH@MX as advanced electrodes for flexible supercapacitors. Carbon.

[115] Zhang W., Qi J., Cao T., Lei Z., Ma Y., Liu H., Zhu L., Feng X., Wei W., Zhang H. (2022). A facile method synthesizing marshmallow ZnS grown on Ti_3_C_2_ MXene for high-performance asymmetric supercapacitors. J. Energy Storage.

[116] Maron G.K., Alano J.H., Noremberg B.S., Rodrigues L.S., Stolojan V., Silva S.R.P., Villarreal Carreño N.L. (2020). Electrochemical supercapacitors based on 3D nanocomposites of reduced graphene oxide/carbon nanotube and ZnS. J. Alloys Compd..

[117] Rani L., Han J.I. (2025). Construction of MWCNT/ZnS/NiS microspheres: Unveiling enhanced electrochemical performance for aqueous asymmetric supercapacitor. J. Energy Storage.

[118] Chu H.-S., Ma D.-M., Wu F., Zhao R.-D., Xiang J., Li L.-F., Zhao X., Wang T. (2025). ZnS@Co(OH)_2_ nanostructured materials with high-performance energy storage and superior electrocatalytic activity. Ceram. Int..

[119] Chavan G.T., Amate R.U., Ahir N.A., Ingole R.S., Mane S.M., An J. (2025). Exploring multi-metallic integration of (Co, Cu, Fe) in ZnS nanostructures: A new paradigm for energy-intensive hybrid supercapacitors. J. Energy Storage.

[120] Ratchatacharoensak A., Jorn-am T., Supchocksoonthorn P., Sirisit N., Chanthad C., Manyam J., Sangtawesin T., Paoprasert P. (2025). Synergistically enhancing the electrochemical performance of activated carbon with ZnS for the fabrication of asymmetric supercapacitors. J. Electroanal. Chem..

[121] Wu H., Liu M., Liu J., Song Y., Sun B., Zhang C., Xu Y., Cao Y., Chen C. (2023). Direct growth of AC/ZnS–Ni_7_S_6_/Ni(OH)_2_ on nickel foam as a porous electrode material for high-performance supercapacitors. Electrochim. Acta.

[122] Shohag M.R.H., Khan M.M.R., Saleh M.A., Supta A.S., Chowdhury M.S.H.H., Rahman M.M., Marwani H.M., Rahman M.M., Okoli O. (2023). Easy fabrication of L-glutamic acid/ZnS composites for efficient photocatalytic and supercapacitor performance. RSC Adv..

